# Correction: A Large Collection of Novel Nematode-Infecting Microsporidia and Their Diverse Interactions with *Caenorhabditis elegans* and Other Related Nematodes

**DOI:** 10.1371/journal.ppat.1006204

**Published:** 2017-02-15

**Authors:** The PLOS Pathogens Staff

In Table 2, dividing lines were introduced during typesetting that affect the readability of the table. The publisher apologizes for the error. Please see the corrected Table 2 here.

**Table 2:** Collection of other microsporidia species infecting wild nematodes

Microsporidia	Nematode host	Strain	Country / Year	Sample composition	Spore feature
*Nematocida* *major* n. sp.	*C*. *briggsae*	**JUm2507**	Thailand / 2013	rotting fruit	2S
	*C*. *briggsae*	JUm2747	Guadeloupe / 2014	rotting fruit	2S
	*C*. *tropicalis*	JUm2751	Guadeloupe / 2014	rotting fruit	n. d.
*Nematocida* *minor* n. sp.	*Oscheius tipulae*	**JUm1510**	Czech Republic / 2008	rotting apple	2S
	*O*. *tipulae*	JUm2772	Armenia / 2014	rotting fruit	n. d.
*Nematocida* *homosporus* n. sp.	*O*. *tipulae*	**JUm1504**	France / 2008	rotting stem	1S
	*Rhabditella typhae*	NICm516	Portugal / 2013	rotting apple	1S
*Nematocida* *ciargi* n. sp.	*Procephalobus* sp.	**JUm2895**	Spain / 2015	rotting fruit	1S
*Nematocida* sp. 7	*C*. sp. 42	**NICm1041**	French Guiana / 2014	flower	n. d.
*Enteropsectra* *longa* n. sp.	*Oscheius* sp. 3	**JUm408**	Iceland / 2002	compost	1S, LT, AP
*Enteropsectra* *breve* n. sp.	*O*. *tipulae*	**JUm2551**	France / 2013	rotting apple	1S, SR, AP
	*O*. *tipulae*	JUm1483	France / 2008	rotting plum	1S, SR,AP
	*O*. *tipulae*	JUm1456	France / 2008	rotting fruit	n. d.
*Pancytospora* *philotis* n. sp.	*O*. *tipulae*	**JUm1505**	France / 2008	rotting apple	LT
	*O*. *tipulae*	JUm1460	France / 2008	rotting snail	LT
	*O*. *tipulae*	JUm1670	France / 2009	rotting apple	LT
	*O*. *tipulae*	JUm2552	France / 2013	rotting stem	LT
*Pancytospora* *epiphaga* n. sp.	*C*. *brenneri*	**JUm1396**	Colombia / 2008	rotting fruit	LT

The reference strain of each newly found species is in bold. 2S: two distinct sizes of spores; 1S: one size of spores

LT: long, thin rod; SR: small rod (see dimensions in Table 3); AP: form spores first along the apical side of the intestinal cells. n. d.: not determined
