# Supplementary figures and images for: A Large Collection of Novel Nematode-Infecting Microsporidia and Their Diverse Interactions with Caenorhabditis elegans and Other Related Nematodes
Source: PLoS Pathog. 2016 Dec 12;12(12):e1006093. doi: 10.1371/journal.ppat.1006093 (PMC5179134; doi:10.1371/journal.ppat.1006093)

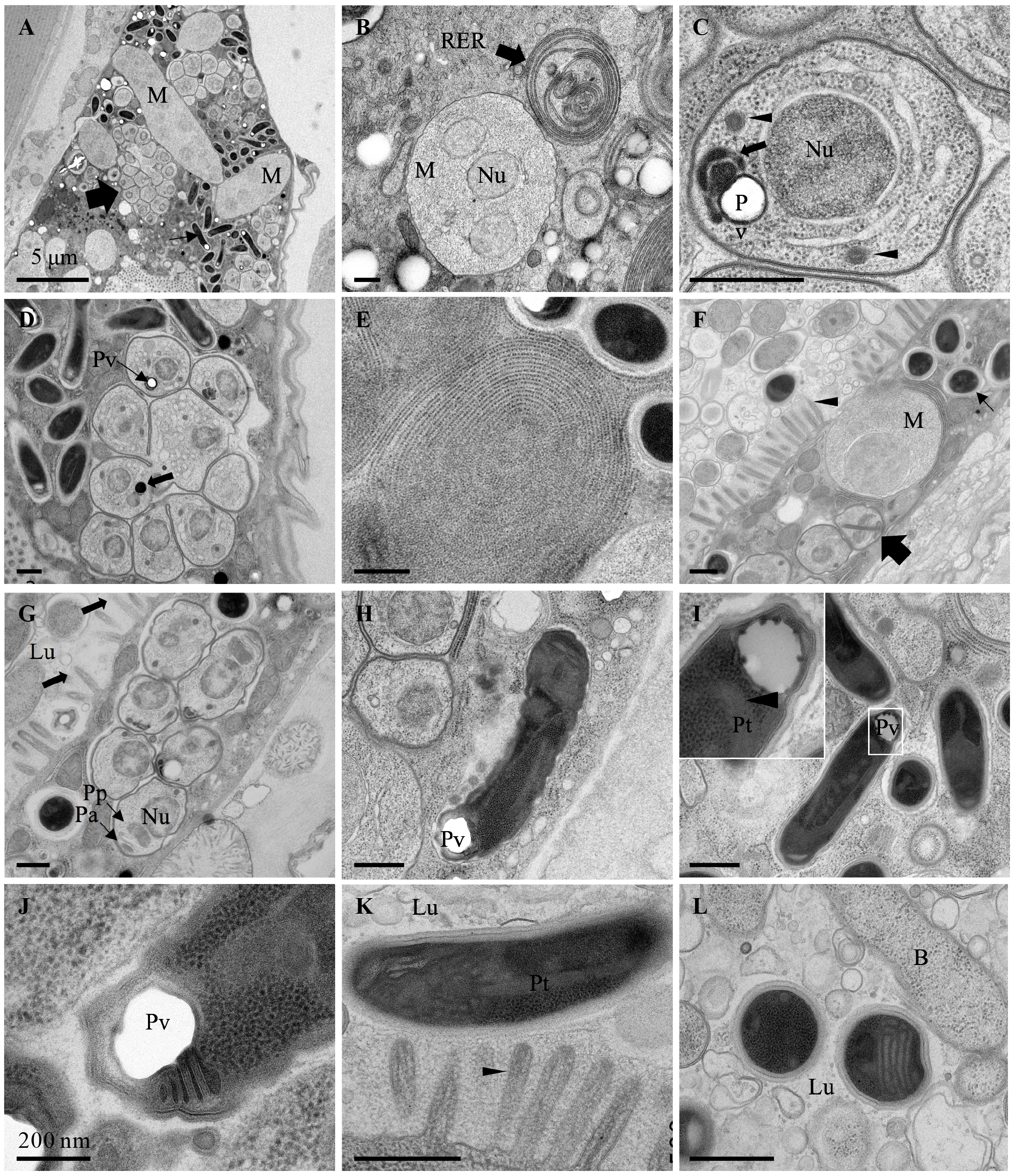

Supplement: S1 Fig — Transmission electron micrographs of N. ausubeli strain JUm2009. A. Lower magnification view of several N. ausubeli infection stages in host intestinal cells (same animal as in Fig 5D). The large arrow indicates sporonts. The small arrow indicates spores. Two multinucleate meronts are indicated. B. A multinucleate meront and a multilayered figure of host rough endoplastic reticulum (arrow). C. Sporont, with a nascent posterior vacuole (arrow) next to the dense membrane apparatus. Arrowheads indicate the nascent polar tube. D. Formation of sporonts in a polysporous sporogon. The small arrow indicates the nascent posterior vacuole and the large arrow indicates the dense membrane apparatus that appears to act as a primordium of the posterior vacuole. E. Rings of host rough endoplastic reticulum. F. Different N. ausubeli infection stages in host intestinal cells. The large arrow indicates sporoblasts. The small arrow indicates spores. One meront with a single nucleus is indicated. Microvilli of host intestinal cell are indicated with arrowhead. G. Six clustered sporonts. The intestinal cell appears reduced in width. The large arrows indicate microvilli; the small arrows indicate nascent anterior and posterior polaroplasts. H. Mature spore with posterior vacuole shown in inset. I. Posterior vacuole in a mature spore. J. Detail of the posterior side of a spore, with the posterior vacuole. K. Mature spore in the lumen, arrowhead indicates microvilli. L. Cross-section of two spores in the intestinal lumen. Scale bar is 500 nm, unless indicated otherwise. B, bacterium; Lu, intestinal lumen; M, meront; Nu, nucleus; Pt, polar tube; Pv, posterior vacuole; RER, rough endoplastic reticulum. (TIF) [file ppat.1006093.s001.tif]

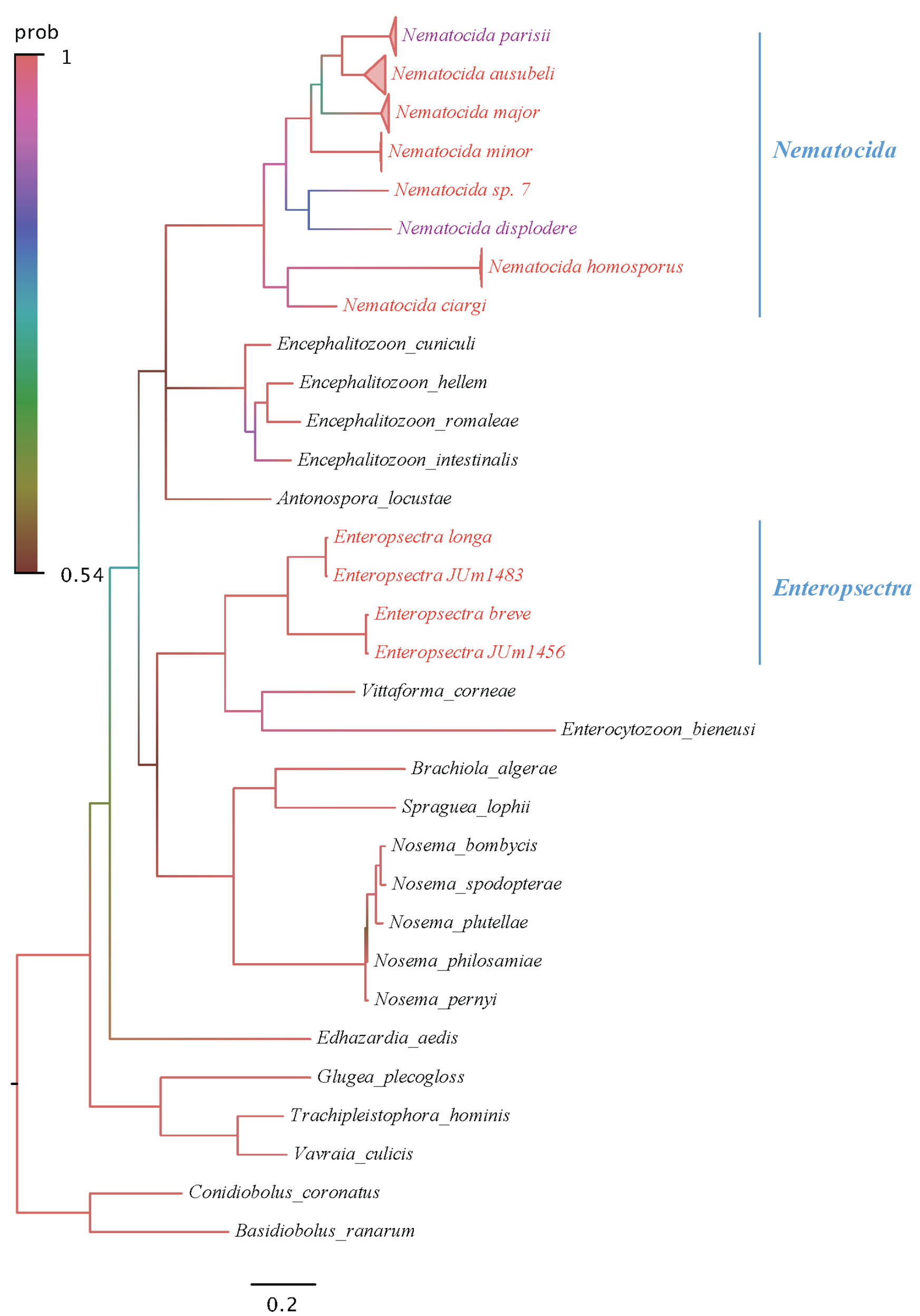

Supplement: S2 Fig — β -tubulin gene sequences from 38 nematode-infecting microsporidia species and 18 other microsporidia species were used. Model General time reversible (GTR) was applied. The branches were colored and annotated as above. This tree shows the position of Nematocida sp. 7. (TIF) [file ppat.1006093.s002.tif]

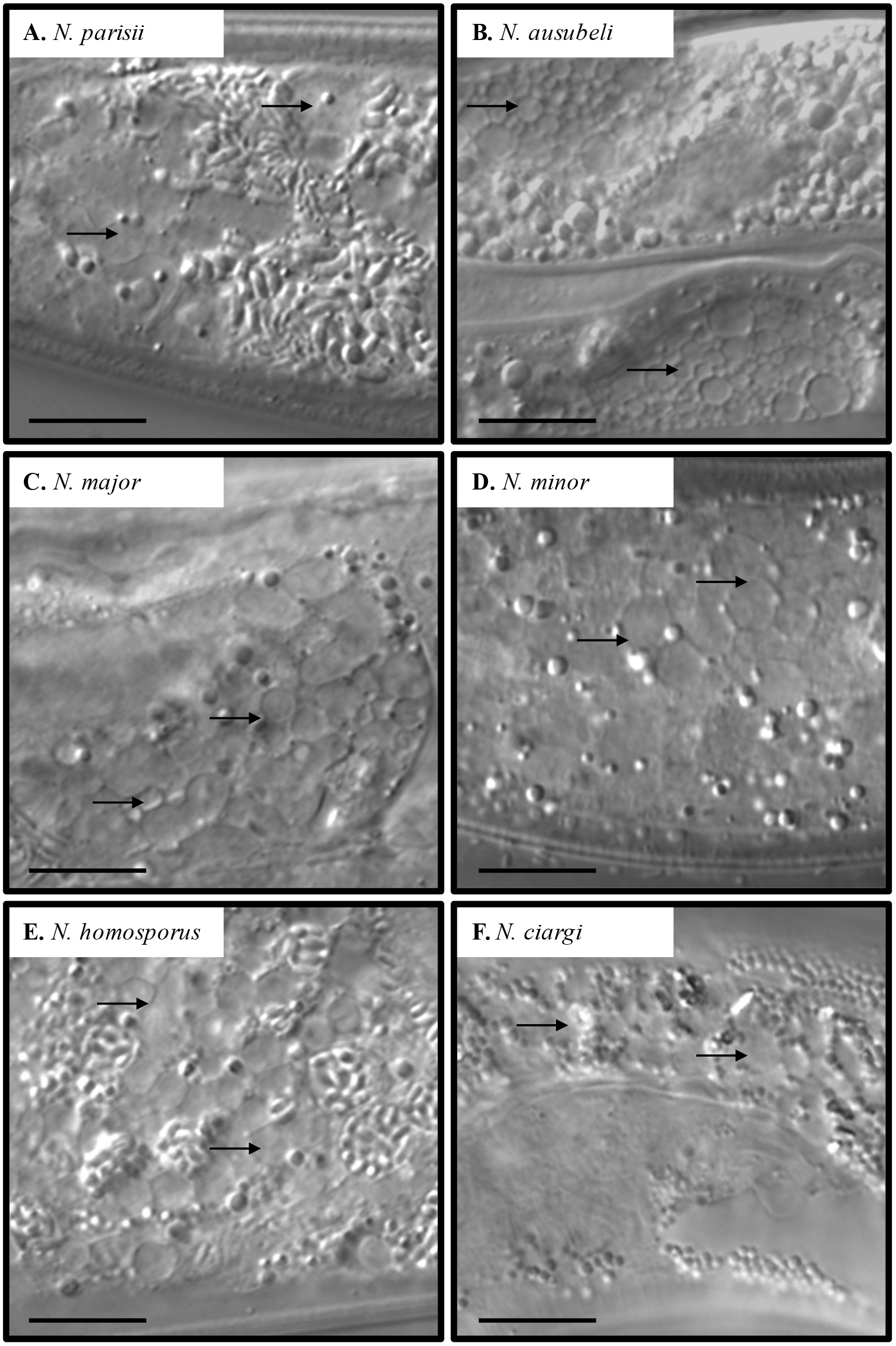

Supplement: S3 Fig — A. Wild Caenorhabditis elegans strain JU1249, with Nematocida parisii infection. B. Wild C. elegans strain JU2520, with Nematocida ausubeli infection. C. Wild C. briggsae stain JU2747, with N. major infection. D. Wild Oscheius tipulae strain JU1510, with N. minor infection. E. Wild Rhabditella typhae strain NIC516, with N. homosporus infection. F. Wild Procephalobus sp. strain JU2895, with N. ciargi infection. Meronts are indicated by arrows in all images. Scale bar: 10 μm (TIF) [file ppat.1006093.s003.tif]

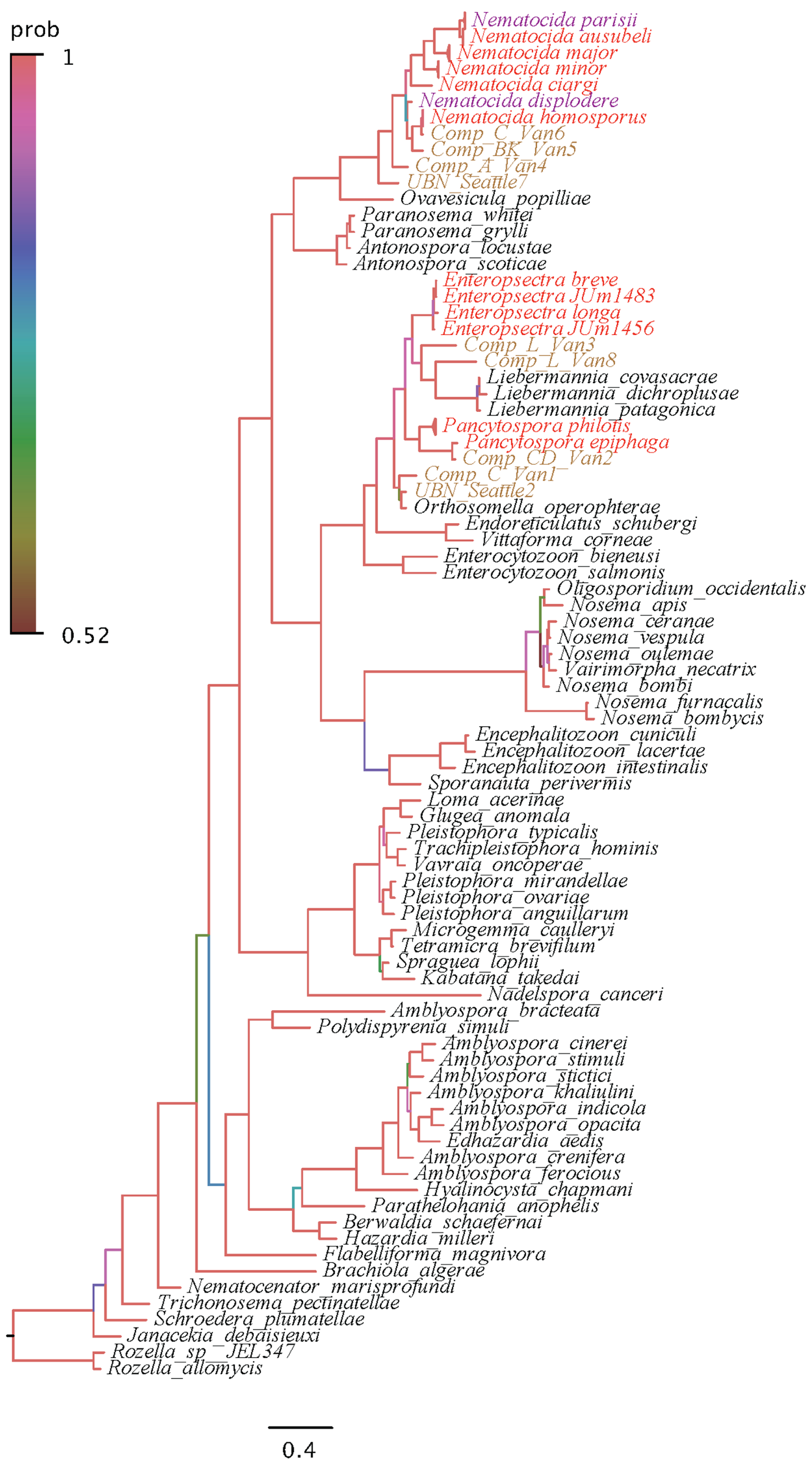

Supplement: S4 Fig — SSU rDNA Bayesian inference phylogeny with the sequences of Fig 2 and nine microsporidian sequences (S1 Table) found in environmental samples in soil, sand and compost samples [41]. Model Kimura 2-Parameter (K2P) was applied. The branches were colored and annotated as Figs 4 & 5. (TIF) [file ppat.1006093.s004.tif]

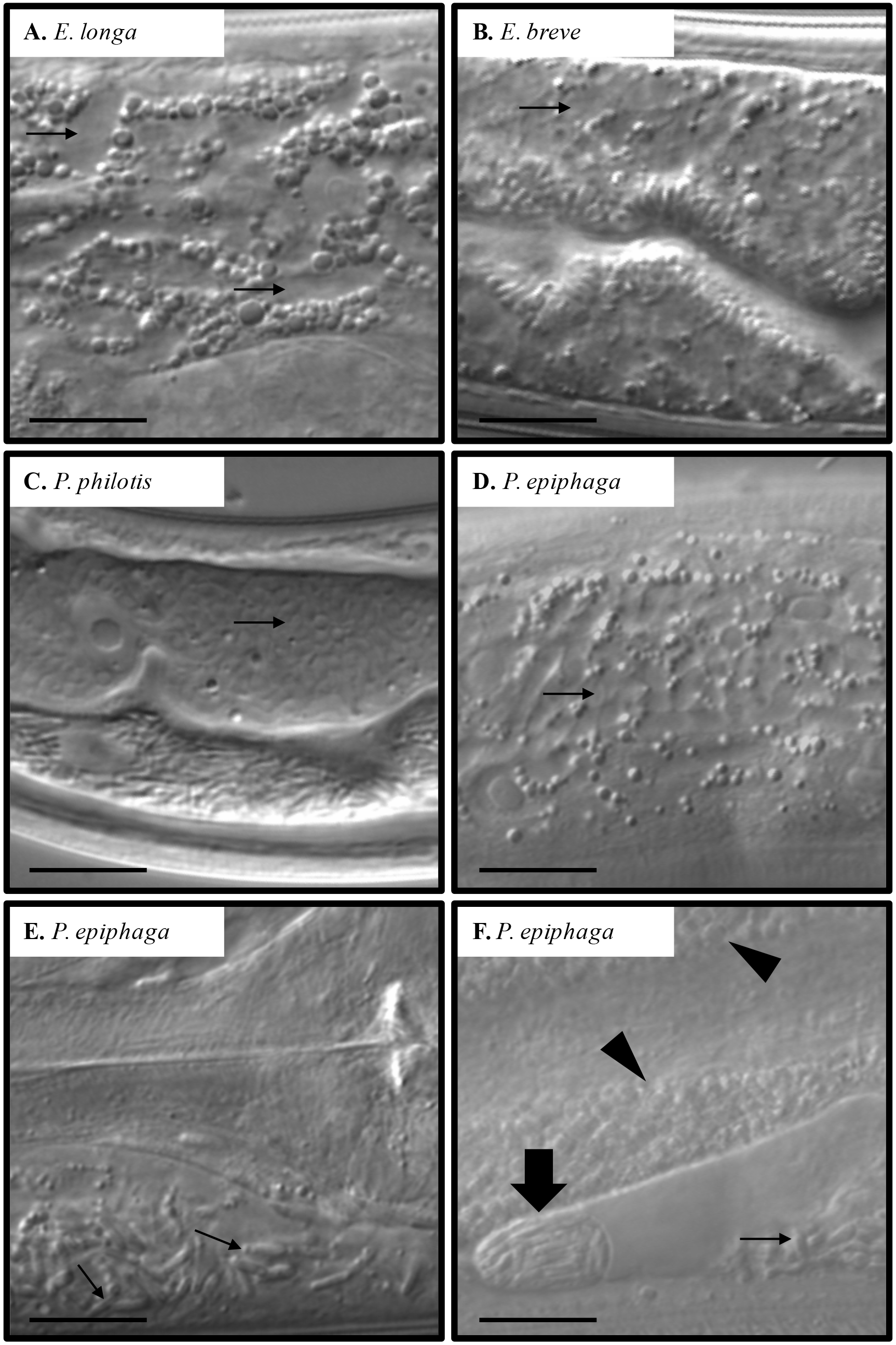

Supplement: S5 Fig — A. Wild Oscheius sp. 3 strain JU408, with Enteropsectra longa infection. B. Wild O. tipulae strain JU2551, with Enteropsectra breve infection. C. Wild O. tipulae strain JU1505, with Pancytospora philotis infection. One intestinal cell (below) was filled with spores, but only meronts seen in the other cell (up) close to it. D. Wild Caenorhabditis brenneri strain JU1396, with Pancytospora epiphaga infection, in the epidermis. Meronts are indicated by arrows in all images. E. Wild Caenorhabditis brenneri strain JU1396, with Pancytospora epiphaga infection in the pharyngeal region. Spores are indicated by arrows. F. Wild Caenorhabditis brenneri strain JU1396, with Pancytospora epiphaga infection. The large arrow indicates spores in the coelomocyte, while the small arrow indicates spores in the epidermis. Arrowheads indicate uninfected intestinal cells that are filled with gut granules. Scale bar: 10 μm (TIF) [file ppat.1006093.s005.tif]

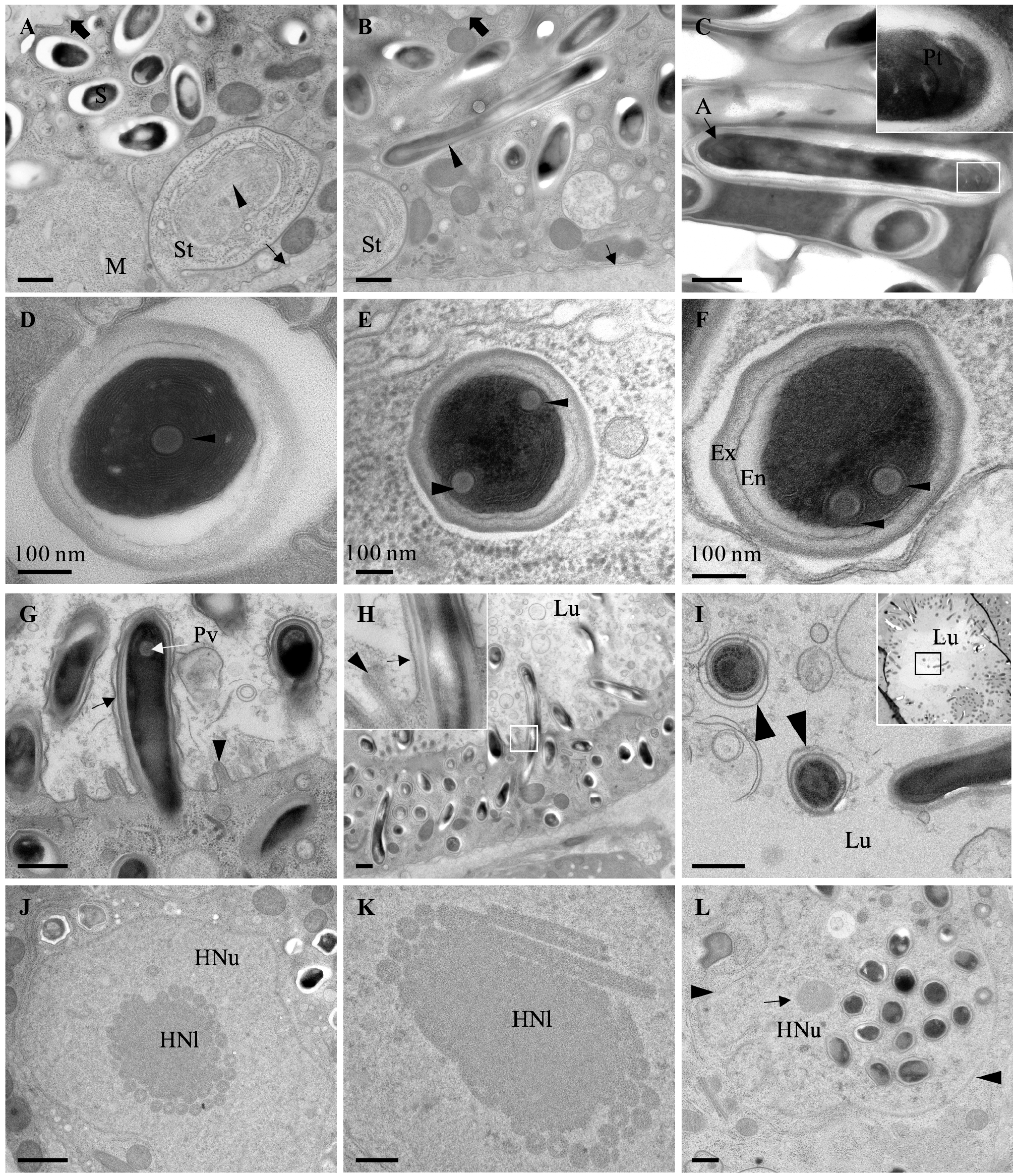

Supplement: S6 Fig — Transmission electron micrographs of E. longa strain JUm408. A, B. Organization of various microsporidian stages in a host cell; the large and small arrows indicate apical and basal membranes of the host intestinal cells, respectively. The spores are on the apical side of the cell (and are not well infiltrated in these sections). In panel A, on the lower right is seen a nuclear division of a sporont. The mitotic spindle is indicated by an arrowhead. In panel B, the longitudinal section of the polar tube of a spore is indicated by an arrowhead. The white halo that can be seen between the mature spore wall and the cytoplasm is not due to the presence of a membrane but to incomplete infiltration during the preparation of the samples for electron microscopy. Such light-appearing areas are also seen occasionally on the internal side of the spore wall in both observed species. C. Mature spore with inset indicating the turn of the polar tube on the posterior end of the spore. Anchoring disk is indicated by arrow. D-F. Cross-section of E. longa spores with arrowheads indicating polar tubes. Most cross-sections show a single section of the polar tube. In E,F are shown the two cases where the polar tube was cut twice, likely close to the posterior end of the spore. G. Exit of spore. G, H. Exit of spores from the intestinal cell apical side into the lumen. The host cell apical membrane (black arrows) folds around E. longa spores. Microvilli are indicated by arrowheads. The posterior vacuole is indicated by a white arrow in panel G. I. Two mature spores are each surrounded by an additional membrane (arrowheads), while the third one (right) does not. Inset at low resolution shows the positions of the two spores in the lumen in the corresponding section. J, K. Host intestinal cell nucleolus. The tubular substructures were not observed in the control uninfected animals. These structures have a width of approximately 250 nm and appear to be formed by ribosomal precursors. L. A nucl [file ppat.1006093.s006.tif]

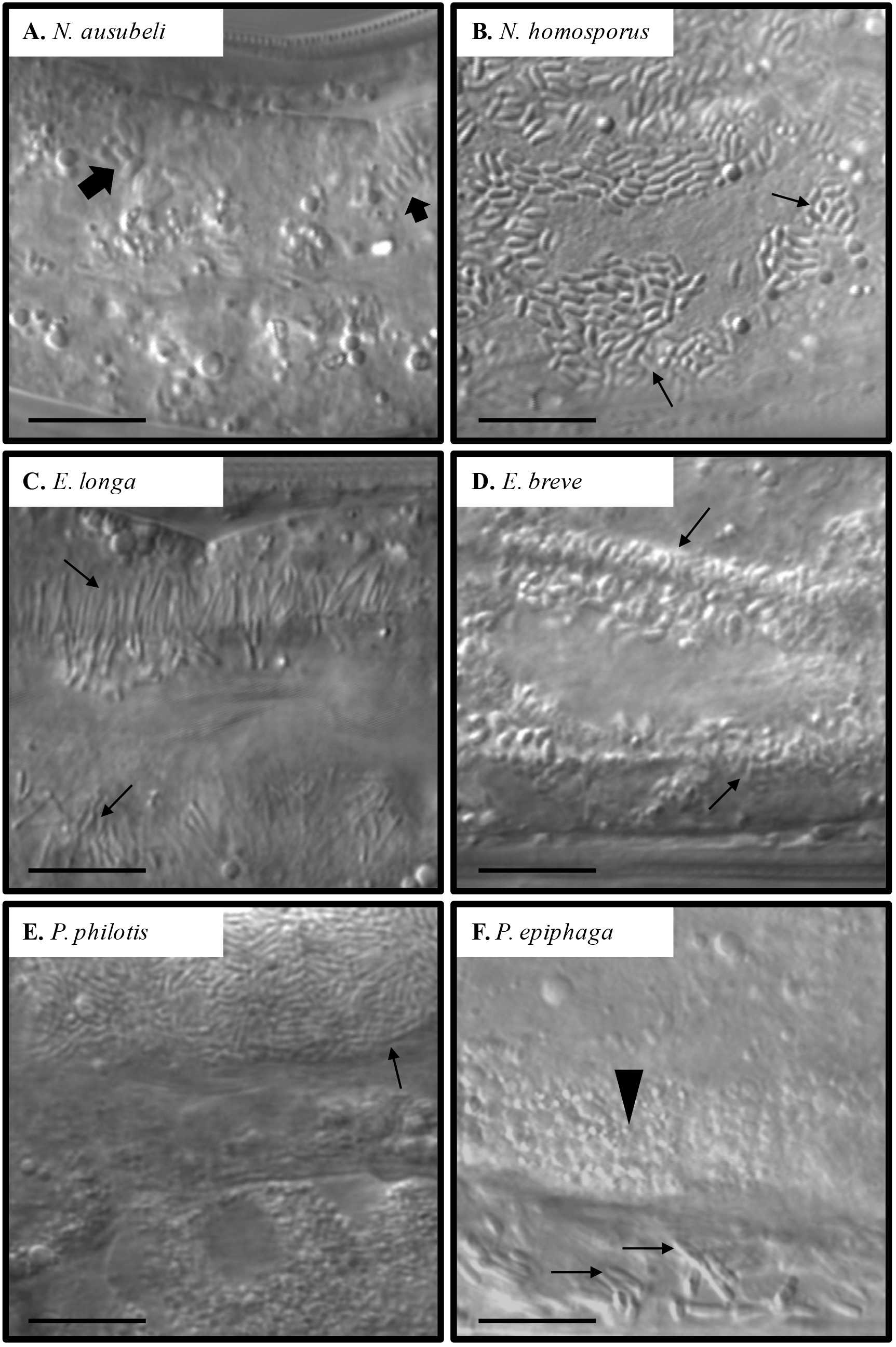

Supplement: S7 Fig — A. Oscheius tipulae strain JU1510, with Nematocida ausubeli JUm2526 infection. Refer to Fig 4B for morphology in the infection of a natural host species. Large and small spore classes are indicated by larger and smaller arrows, respectively. B. Oscheius sp. 3 strain JU75, with N. homosporus NICm516 infection. Refer to Fig 4E for morphology in a natural host species. Spore vesicles are indicated by arrows. C. Oscheius sp. 3 strain JU75, with Enteropsectra longa JUm408 infection. Refer to Fig 7A for morphology in a natural host species. Long thin spores along the apical membrane of host intestinal cells are indicated by arrows. D. Oscheius sp. 3 strain JU408, with Enteropsectra breve JUm2551 infection. Refer to Fig 7B for morphology in a natural host species. Small rod spores along the apical membrane of host intestinal cells are indicated by arrows. E. Oscheius tipulae strain JU170, with Pancytospora philotis JUm1505 infection. Refer to Fig 7C for morphology in a natural host species. Long thin spores concentrated in intestinal cells is indicated by arrow. F. C. elegans N2, with Pancytospora epiphaga JUm1396 infection. The arrowhead indicates intestinal granules. Refer to Fig 7D for morphology in a natural host species. Scale bar: 10 μm. (TIF) [file ppat.1006093.s007.tif]
